# The interaction between muscle pathophysiology, body mass, walking speed and ankle foot orthosis stiffness on walking energy cost: a predictive simulation study

**DOI:** 10.1186/s12984-023-01239-z

**Published:** 2023-09-07

**Authors:** N. F. J. Waterval, M. M. van der Krogt, K. Veerkamp, T. Geijtenbeek, J. Harlaar, F. Nollet, M. A. Brehm

**Affiliations:** 1 Amsterdam UMC Location University of Amsterdam, Rehabilitation Medicine, Meibergdreef 9, Amsterdam, The Netherlands; 2 Amsterdam UMC Location Vrije Universiteit Amsterdam, Rehabilitation Medicine, De Boelelaan 1117, Amsterdam, The Netherlands; 3 Amsterdam Movement Sciences, Rehabilitation and Development, Amsterdam, The Netherlands; 4 School of Health Sciences and Social Work, Griffith University, Gold Coast, Australia; 5 Griffith Centre of Biomedical and Rehabilitation Engineering (GCORE), Menzies Health Institute Queensland, and Advanced Design and Prototyping Technologies Institute (ADAPT), Griffith University, Gold Coast, Australia; 6 Department of Biomechanical Engineering, Delft University of Technology, Delft, The Netherlands; 7 Department of Orthopaedics, Erasmus Medical Center, Rotterdam, The Netherlands

## Abstract

**Background:**

The stiffness of a dorsal leaf AFO that minimizes walking energy cost in people with plantarflexor weakness varies between individuals. Using predictive simulations, we studied the effects of plantarflexor weakness, passive plantarflexor stiffness, body mass, and walking speed on the optimal AFO stiffness for energy cost reduction.

**Methods:**

We employed a planar, nine degrees-of-freedom musculoskeletal model, in which for validation maximal strength of the plantar flexors was reduced by 80%. Walking simulations, driven by minimizing a comprehensive cost function of which energy cost was the main contributor, were generated using a reflex-based controller. Simulations of walking without and with an AFO with stiffnesses between 0.9 and 8.7 Nm/degree were generated. After validation against experimental data of 11 people with plantarflexor weakness using the Root-mean-square error (RMSE), we systematically changed plantarflexor weakness (range 40–90% weakness), passive plantarflexor stiffness (range: 20–200% of normal), body mass (+ 30%) and walking speed (range: 0.8–1.2 m/s) in our baseline model to evaluate their effect on the optimal AFO stiffness for energy cost minimization.

**Results:**

Our simulations had a RMSE < 2 for all lower limb joint kinetics and kinematics except the knee and hip power for walking without AFO. When systematically varying model parameters, more severe plantarflexor weakness, lower passive plantarflexor stiffness, higher body mass and walking speed increased the optimal AFO stiffness for energy cost minimization, with the largest effects for severity of plantarflexor weakness.

**Conclusions:**

Our forward simulations demonstrate that in individuals with bilateral plantarflexor the necessary AFO stiffness for walking energy cost minimization is largely affected by severity of plantarflexor weakness, while variation in walking speed, passive muscle stiffness and body mass influence the optimal stiffness to a lesser extent. That gait deviations without AFO are overestimated may have exaggerated the required support of the AFO to minimize walking energy cost. Future research should focus on improving predictive simulations in order to implement personalized predictions in usual care.

*Trial Registration* Nederlands Trial Register 5170. Registration date: May 7th 2015. http://www.trialregister.nl/trialreg/admin/rctview.asp?TC=5170

**Supplementary Information:**

The online version contains supplementary material available at 10.1186/s12984-023-01239-z.

## Introduction

Individuals with plantarflexor weakness caused by neuromuscular disorders walk with excessive ankle dorsiflexion, persistent knee flexion and a reduced push-off during stance [1, 2]. These gait deviations cause an increase in metabolic walking energy cost [3–5], limiting daily-life physical mobility.

To improve the gait pattern and reduce walking energy cost, a dorsal leaf spring ankle–foot orthosis (AFO) can be provided [6, 7]. In case of plantarflexor weakness, such an AFO should provide an external plantarflexion moment during stance that is large enough to prevent excessive ankle dorsiflexion. This way, the ground reaction force vector can move anterior of the ankle and in front of the knee [6, 8], allowing the knee to extend. Additionally, ankle push-off power can be supported by storing energy in the dorsal leaf spring when the ankle moves towards dorsiflexion and releasing this energy when the ankle moves towards plantarflexion [9–11]. This energy can take over work of the plantarflexors [10] and/or reducing ipsilateral hip or contralateral leg compensations [12, 13]. Both extending the knee and support of ankle push-off power contribute to the reduction in walking energy cost [10, 14]

To maximize the AFO’s effect on walking energy cost reduction, the AFO’s bending stiffness needs to be individually optimized [15–19]. A very flexible AFO will not provide a large enough external plantarflexion moment to decrease ankle dorsiflexion and extend the knee, while a rigid AFO impedes ankle push-off power [7, 17, 18]. Consequently, the stiffness that maximally reduces walking energy cost is the best trade-off between flexibility and rigidity, which differs between individuals.

What factors affect the individual optimal stiffness is not completely understood [15, 20]. Muscle pathophysiology, such as severity of plantarflexor weakness and passive stiffness, and also body mass likely influence the degree of AFO bending stiffness to best normalize joint kinematics [20, 21] and minimize walking energy cost. Moreover, walking speed affects the strain on the AFO [21] and as demonstrated by a conceptual hip torque-driven model also influences the optimal AFO stiffness for energy cost minimization [18]. However, how muscle pathophysiology, body mass, walking speed and the optimal stiffness for energy cost minimization interact and whether this optimal AFO stiffness coincides with the AFO stiffness best normalizing joint kinematics and kinetics has not been studied.

By means of human experiments, these interactions are challenging to study, as systematic variations cannot be imposed in most cases and/or require extensive testing. Forward dynamic predictive simulations are not limited in this regard and have shown their potential by validly predicting the effects of bilateral plantarflexor muscle weakness on gait [22]. Yet, whether these methods can also predict the effects of assistive devices, and specifically AFOs, on gait warrants further exploration. Our goals in the present study were to (1) validate a forward-dynamic predictive simulation framework to predict the effect of dorsal leaf AFOs on gait in people with bilateral plantarflexor weakness, (2) study the effects of muscle pathophysiology (i.e. plantarflexor weakness and passive stiffness), body mass, walking speed and their interaction with the optimal AFO stiffness for energy cost minimization, and (3) evaluate whether the optimal stiffness for energy cost minimization corresponds to the stiffness best normalizing joint kinematics and kinetics.

## Methods

To generate walking simulations, a forward simulation framework consisting of a reflex-based controller and a neuromuscular model with plantarflexor weakness and an AFO was created. First, we validated this framework against previously collected gait data of people with bilateral plantarflexor weakness walking with and without dorsal leaf spring AFO’s [23]. Secondly, we performed forward simulations with different AFO stiffness configurations for models in which muscle pathophysiology, body mass and walking speed were systematically varied. Third, we evaluated whether the optimal stiffness for energy cost minimization coincided with the stiffness best normalizing joint kinematics and kinetics.

### Forward simulation framework

We used a previously developed musculoskeletal model, consisting of seven segments (trunk-pelvis, and left and right thigh, shank and foot), nine degrees of freedom, and nine musculotendon units on each leg modelled with the Millard Equilibrium muscle model [24]. The following muscles were included: Tibialis Anterior, Soleus, Gastrocnemius medialis, Vastus intermedius, Rectus Femoris, Semitendinosus, Biceps Femoris short head, Gluteus Maximus and Iliopsoas, each representing the associated muscle group. Peak isometric forces, mass of the segments, muscle paths, optimal fiber length, pennation angle and tendon slack length were set according to our previous simulation work in bilateral plantar flexor weakness and based on the OpenSim Gait2392 model [25]. Knee ligaments were modelled as a rotational spring (2 Nm/degree) and damper (0.2 Nm/degree/s)), which were activated when the knee moved beyond 120 degrees of flexion or extended beyond 10 degrees of flexion. Ground contact was modelled by two viscoelastic Hunt-Crossley contact spheres on each foot, based on Veerkamp et al. [26, 27]. An overview of all model parameters can be found in Additional file 1: Appendix A. The baseline model was converted into a Hyfydy (https://hyfydy.com [28]) model using SCONE software, which is an open-source optimization toolbox [29]. Hyfydy musculoskeletal models are specifically designed for forward simulations, and allow for much faster optimizations compared to OpenSim models. Details on the differences between the original OpenSim model and the developed Hyfydy model can be found in Additional file 2: Appendix B.

To activate the muscles, a gait state-dependent reflex-based controller was used [22], which was adapted from Geyer & Herr [30]. In short, the controller consisted of a combination of constant signals and force- and length-based reflexes that could change between gait phases. For trunk stabilization, the hamstrings, iliopsoas and gluteus maximus muscles were also activated by a proportional-derivative feedback loop.

Reflex gains within each gait phase, transition thresholds between the phases and the initial joint angles were optimized by minimizing a cost function using the Covariance Matrix Adaptation Evolution Strategy (CMA-ES) [31], as implemented in SCONE [29]. Based on previous work, the cost function consisted of walking energy cost, head acceleration, walking without falling down, and avoiding unrealistic knee and ankle angles [22, 27]. Furthermore, to better simulate the effects of plantar flexion weakness by avoiding unrealistic high activations of the weakened muscles, muscle activation above 50% of the plantarflexor muscles was penalized. Such activations would cause rapid muscle fatigue and are biologically unlikely [32, 33]. We choose to apply a penalty on high muscle activation instead of muscle activation squared, as a muscle activation squared penalty reduces walking speed [27]. Weightings of the different components were set as follows: $0.15*\text{Walking energy cost}+0.1*\text{Head Acceleration}+1E8*\text{avoid falling down}+0.1*\text{unrealistic joint angles}+1E4*\text{activation above}\,50\%\,\text{of plantarflexors}$. In the final simulation outcomes, only walking energy cost and head acceleration contributed to the score of the cost function.

Walking was simulated for bouts of 10 s with a minimum walking speed of 0.3 m/s. We optimized each simulation six times with different random seeds, as the optimization algorithm is based on random alterations. The optimization was terminated when, averaged over the last 500 simulation generations, the improvement in cost function outcome was smaller than 0.001%. The best of the six optimizations was selected as outcome.

To impose plantarflexor weakness, the isometric force of the Soleus and Gastrocnemius muscles was reduced, while passive muscle and tendon stiffness were adapted such that passive fibre and tendon force–length curves matched those of the unimpaired model [22]. The AFO was modelled as a torsional massless spring around both ankles with a neutral angle of zero degrees.

### Validation of the forward simulation framework

For validation of the framework, we first performed simulations for a model without weakness, our model with 80% plantarflexor weakness without and with an AFO with a stiffness of 2.6 Nm/degree. The simulations were compared with the average experimental 3D gait data of 11 participants of the PROOF-AFO trial with bilateral plantarflexor weakness and manually tested maximal ankle dorsiflexion angle of at least zero [23]. These participants had on average an 80% lower maximal isometric force was measured on a fixed dynamometer (Biodex) compared to our norm dataset [22]. Characteristics of the participants are presented in Table 1 [17, 23]. 3D gait data for walking with shoes only and with an AFO of 2.6 Nm/ degree were measured at comfortable, self-selected speed using an 8-camera 100 Hz Vicon MX 1.3 system (VICON, Oxford, UK) and four force plates (1000 Hz, OR6-7, AMTI, Watertown, USA). Markers were placed according to the Plug-In-Gait model [34], and processed in OpenSim to calculate sagittal lower limb joint angles and moments [35] and derived joint powers. The joint angles, moments and powers were time-normalized to one gait cycle using custom-made scripts in Matlab.

**Table 1:** Characteristics of the participants

Gender (male/female)	5/6
Age in years	55.7 ± 16.3
Weight in kilogram	89.6 ± 15.1
Manual muscle score in median (range)	Knee extension: 5 (5–4.5) Ankle plantarflexion: 4 (5–1) Ankle dorsiflexion: 2 (4.5–0)
Maximal ankle dorsiflexion manually tested in supine position in degrees	4.1 ± 6.3
Diagnosis	Charcot-Marie-Tooth disease (n = 7) Poliomyelits (n = 2) Myotonic dystrophy (n = 1) Myoshi myopathy (n = 1)

For validation, the agreement between the simulations with plantarflexor weakness without and with AFO and experimental data was quantified by time-normalized cross-correlations (R) and the root mean square error (RMSE) normalised to the standard deviation (SD) for sagittal lower limb joint angles, moments and powers. An RMSE below 2 standard deviations (SD) means that on average the simulation was within the 95% confidence interval of the experimental data. Additionally, to test in which time periods within the gait cycle the simulations differed significantly from the experimental data, an independent t-test was performed over the whole gait cycle using statistical parameter mapping (SPM, version M.0.4.8) [36].

### Simulation of effects of muscle pathophysiology, body mass and speed on optimal AFO stiffness

Next, we created models in which muscle pathophysiology and body mass were systematically varied. For each model, we performed simulations for walking without AFO (stiffness 0 Nm/degree) and with AFO with stiffness levels ranging from 0.9 Nm/degree, being very flexible, to 8.7 Nm/degree, being very stiff, with increments of 0.87 Nm/degree. This range coincides with the range of stiffness levels typically used in clinical practice [7, 19, 37]. To study the effect of severity of plantarflexor weakness, models with a bilateral imposed reduction in plantarflexor strength of 40%, 60% and 90% were created, besides our model with an 80% reduction in strength used for the validation. Using the 80% weakness model, the passive plantarflexor stiffness was varied irrespective of weakness, to be 20%, 50%, 150% and 200% of normal, which is approximately the range found in healthy individuals [38]. To study the effects of body mass, we increased the mass of the segments by 10% and 30%, without changing muscle forces indicating less force per kg as seen in obese subjects [39], again using the model with 80% weakness. In these simulations walking speed was an optimization parameter and therefore could differ between models and conditions.

To systematically evaluate the effects of walking speed we simulated walking with the baseline 80% weakness model with the speed set at 0.75, 1.0 and 1.2 m/s, which represents the range of walking speeds clinically seen in these patients [7]. Additionally, simulations at a set speed of 1.2 m/s for the models with 40% and 90% plantarflexor weakness, 20% and 200% of normal passive plantarflexor stiffness and with body mass increased by 30% were performed to study the interaction between these factors and walking speed. For the combinations that showed no optimum until 8.7 Nm/degree, we extended the simulated AFO stiffness range to 12.2 Nm/degree.

For each model, the optimal stiffness for walking energy cost reduction was determined by the minimum (e.g. point were energy cost is maximally reduced) of a 3rd order polynomial fit across the eleven simulations, i.e. without AFO (stiffness 0) and with AFO for 10 stiffness levels (Fig. 2). A polynomial fit with a correlation coefficient above 0.7 was considered good [40]. A 3rd order fit was drawn as it cannot be assumed that the slope of walking energy cost versus AFO stiffness is symmetric left and right of the minimum.

### AFO stiffness best normalizing gait

To study whether the optimal stiffness for energy cost minimization coincided with the stiffness best normalizing joint kinematics and kinetics, a 3^rd^ order best polynomial fit was drawn between AFO stiffness and the maximal ankle angle, maximal ankle moment, minimal knee angle and minimal external knee moment. The stiffnesses where the fit equalized the mean normative value for maximal ankle angle (angle of 17.2 degrees), minimal knee angle (4.2 degrees) and external knee extension moment (below 0.34 Nm/kg) during stance were calculated. For maximal ankle moment the mean normative value minus 2 SD was used (1.17 Nm/kg), as no stiffness reached the mean normative value and ankle moment levelled off at high stiffness levels (Fig. 2). These four stiffness values that best normalized the specific gait outcomes were compared to optimal stiffness for energy cost minimization with independent t-tests, in which each simulation model was regarded an individual subject.

## Results

### Validation of the forward simulation framework

**Fig. 1:**
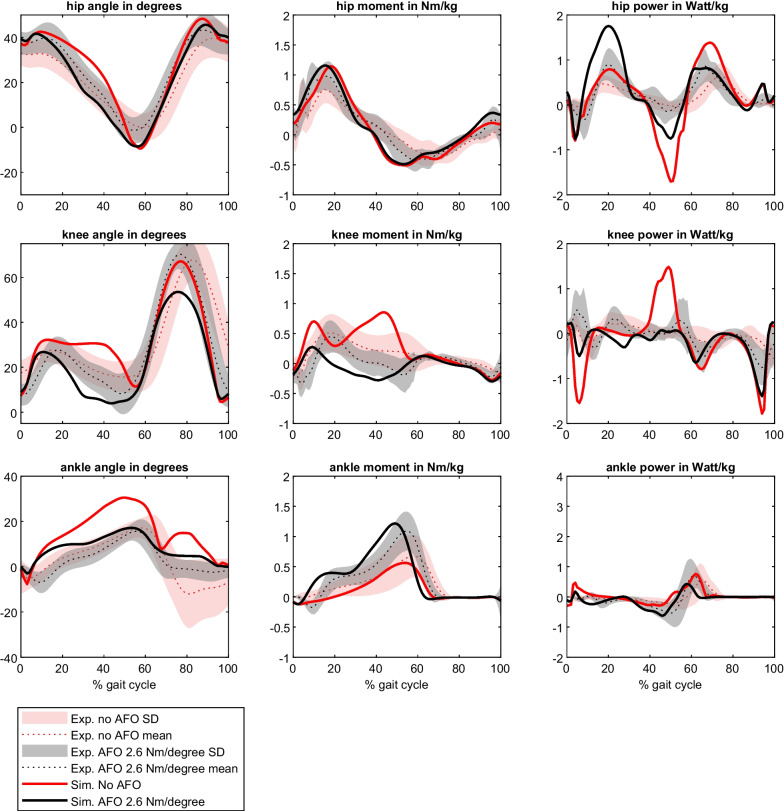
Comparison of simulations of a model without muscle weakness, and model with 80% plantarflexor weakness without and with AFO (2.6 Nm/degree) with experimental data of people with bilateral plantarflexor weakness walking with and without AFO

**Fig. 2:**
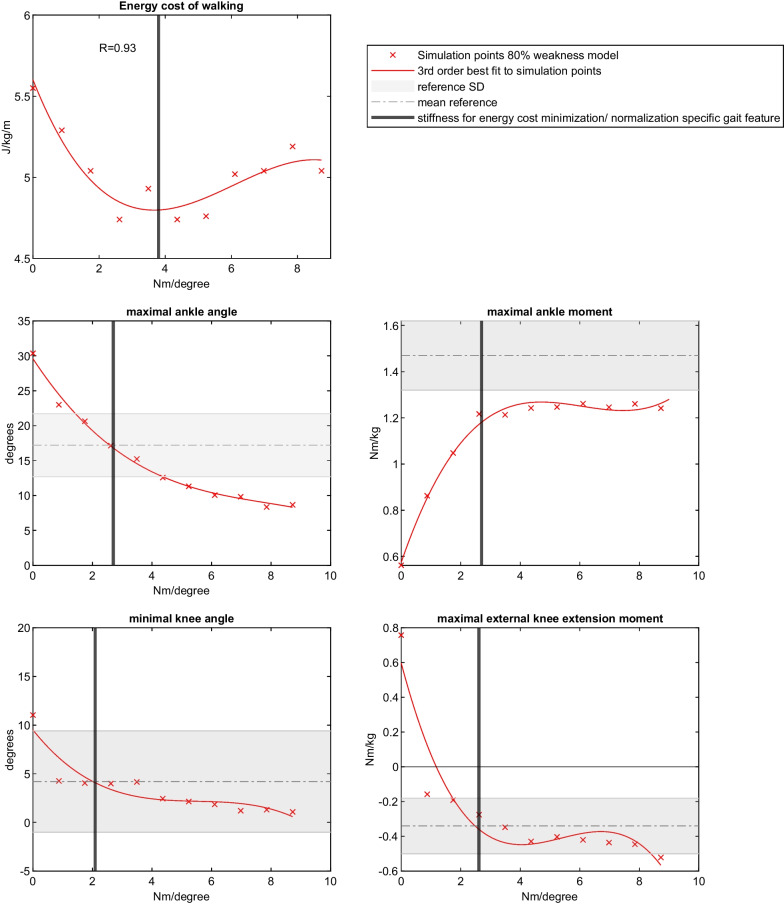
Example of how optimal stiffness for energy cost minimization and stiffness necessary for gait parameter normalization was determined

The results of our simulation without muscle weakness can be found in Additional file 3. For the 80% weakness model walking without AFO, cross-correlation between simulation and experimental data was above 0.64 for all joint angles, moment and powers. The RMSE values of all angles and moments were within 2 SD, although the simulated ankle dorsiflexion angle was significantly higher during most part of the stance phase. For joint powers, only the ankle power showed an RMSE within 2 SD, while simulated knee power (RMSE 3.22 SD) and hip power (RMSE 3.52 SD) deviated more from experimental data and demonstrated significantly higher power peaks compared to the experimental data. SPM analysis demonstrated that mainly the ankle angle different significantly during stance (18–52% of gait cycle), while for the other parameters only short periods were significant. For walking with AFO with a stiffness of 2.6 Nm/degree, all joint angles, moments and powers demonstrated strong cross-correlations, except the knee moment (R = 0.54), with RMSEs were all within 2 SD and there were only short periods of the gait cycle with significant differences between simulations and experimental data (Figs. 1, 2, Table 2 and Additional file 4: Appendix D).

**Table 2:** Cross-correlation and Root-Mean-Square error for walking without AFO and with AFO with a stiffness of 2.6 Nm/degree

	Walking without AFO			Walking with AFO of 2.6 Nm/degree		
	R	RMSE in SD	% GC different (SPM)	R	RMSE in SD	% GC significant SPM
Ankle angle	0.64*	1.98	18–52% GC	0.83	1.26	–
Knee angle	0.96	1.54	–	0.98	1.11	72–75% GC
Hip angle	0.98	1.23	28–34% GC	0.98	0.73	–
Ankle moment	0.95	0.80	1–3%, 98–100% GC	0.99	1.78	1–3% GC
Knee moment	0.84	1.31	6–9% GC	0.54*	0.87	–
Hip moment	0.92	1.34	17–25% GC	0.96	1.60	–
Ankle power	0.84	1.49	1–8% GC	0.90	1.03	1–3% GC
Knee power	0.65*	3.22*	3–9% GC	0.70	1.24	26–29% GC
Hip power	0.73	3.52*	3–6%, 42–54%, 71–80% GC	0.89	1.93	12–18%, 48–50%, 85–89% GC

*AFO* ankle foot orthosis, R cross-correlation coefficient, *RMSE* root-mean-square error, *SD* standard deviations, *GC* Gait cycle, *SPM* statistical parameter mapping

*is considered a moderate cross-correlation (R = 0.5–0.7) or high RMSE (> 2.0 SD)

### Effects of muscle pathophysiology, body mass, walking speed and optimal AFO stiffness

**Fig. 3:**
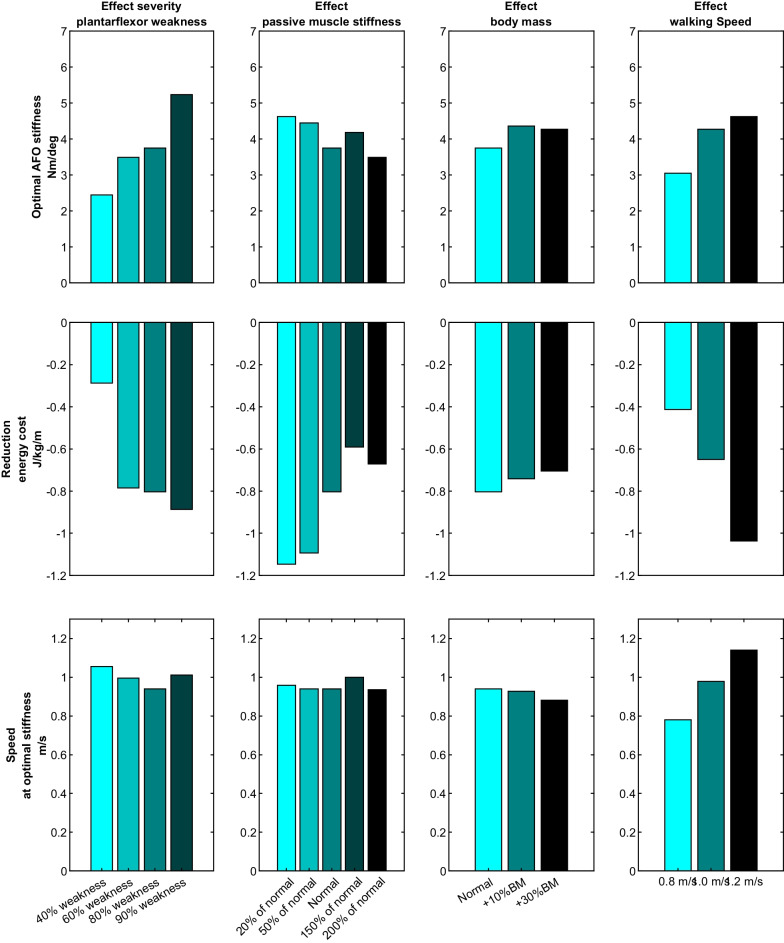
Effects of muscle pathophysiology, body mass and walking speed on the optimal AFO stiffness and reduction in energy cost

A strong 3rd order polynomial fit between AFO stiffness and energy cost was found for all models (r > 0.77). With a larger reduction in plantarflexor strength, the optimal stiffness increased from 2.4 Nm/degree at 40% plantarflexor weakness to 5.2 Nm/degree at 90% weakness, coinciding with a higher reduction in energy cost (from 0.29 to 0.89 J/kg/m) (Fig. 3). A reduction in passive plantarflexor stiffness resulted in slightly higher optimal AFO stiffness levels (3.7 with normal passive stiffness to 4.5 Nm/degree with 20% of normal) and a higher reduction in energy cost (from 0.68 to 1.15 J/kg/m). With a 10% higher body mass, the optimal stiffness increased from 3.7 Nm/degree to 4.4 Nm/degree, but reduced back to 4.3 Nm/degree at 30% higher body mass. Also, the reduction in walking energy cost decreased with higher body mass from 0.80 to 0.70 J/kg/m. Increasing walking speed from 0.75 m/s to 1.2 m/s increased the optimal stiffness from 3.0 to 4.6 Nm/degree, with a reduction in energy cost from 0.41 to 1.03 J/kg/m.

At the fixed speed of 1.2 m/s, the difference in optimal stiffness between the various severities of plantarflexor weakness reduced. The optimal stiffness increased from 2.9 Nm/degree with 40% plantarflexor weakness to only 3.8 Nm/degree with 90% weakness. At this fixed speed, no substantial difference in optimal stiffness between the low and high passive plantarflexor stiffness models was found, while the optimal AFO stiffness with a 30% higher body mass increased from 4.3 Nm/degree to 7.1 Nm/degree.

### AFO stiffness best normalizing gait

**Fig. 4:**
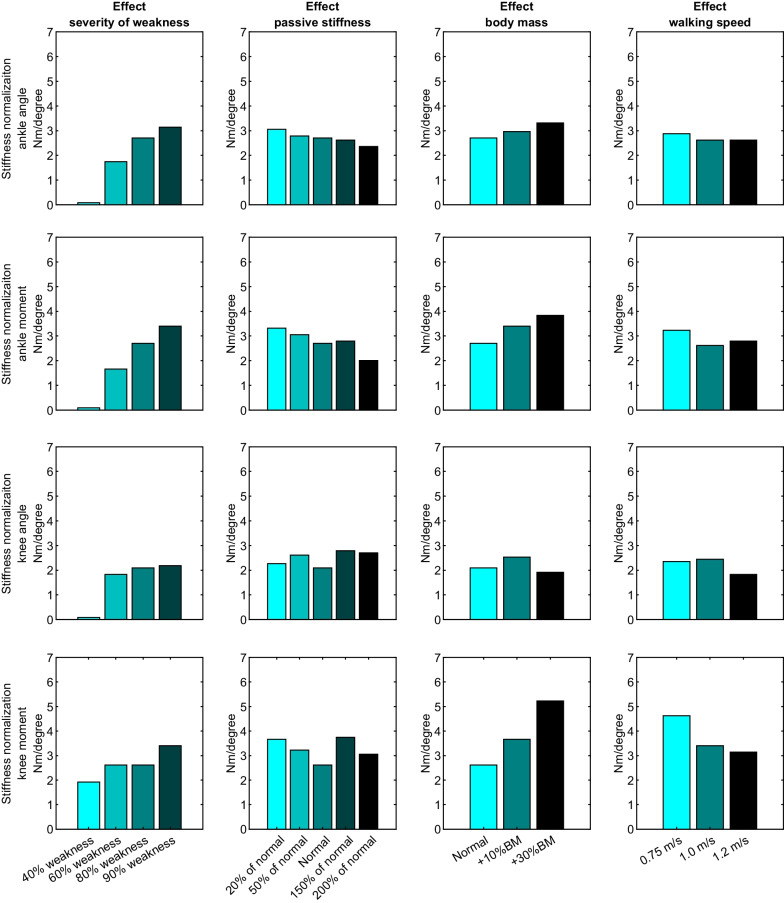
Effects of muscle pathophysiology, body mass and walking speed on the minimum AFO stiffness necessary to normalize the ankle angle, ankle moment, knee angle and knee moment

The average optimized stiffness for walking energy cost minimization over all models (4.3 ± 0.8 Nm/degree) was higher compared to the average stiffness resulting in a normalized maximal ankle angle (mean difference: 1.9 ± 0.9 Nm/degree, *p* < 0.001), ankle moment (mean difference: 1.6 ± 1.0 Nm/degree, *p* < 0.001), knee angle (mean difference: 2.4 ± 1.4 Nm/degree, *p* < 0.001) and knee moment (mean difference: 1.1 ± 0.9 Nm/degree, *p* < 0.001). To best normalize the ankle angle and moment, a higher AFO stiffness was needed with more severe plantarflexor weakness, higher body mass and lower levels of passive plantarflexor stiffness (Fig. 4). Regarding the knee angle, the model with 40% weakness had a normal knee flexion angle without AFO, while in all other models the minimum stiffness that normalized the knee angle was between 1.8 and 2.8 Nm/degree (Fig. 4, Table 3). To best normalize the knee moment, a higher AFO stiffness was needed with more severe weakness and higher body mass.

**Table 3:** Outcome parameters of all different musculoskeletal models

Model	Optimal AFO stiffness in Nm/degree	Reduction in energy cost in J/kg/m	Walking speed with optimal AFO stiffness in m/s	Minimum AFO stiffness for normalization ankle angle in Nm/degree	Minimum AFO stiffness for normalization ankle moment in Nm/degree	Minimum AFO stiffness for normalization knee angle in Nm/degree	Minimum AFO stiffness for normalization knee moment in Nm/degree
Baseline	@ optimized speed / at fixed speed of 1.2 m/s	@ optimized speed / at fixed speed of 1.2 m/s	@ optimized speed / at fixed speed of 1.2 m/s	@ optimized speed / at fixed speed of 1.2 m/s	@ optimized speed / at fixed speed of 1.2 m/s	@ optimized speed / at fixed speed of 1.2 m/s	@ optimized speed / at fixed speed of 1.2 m/s
80% weakness	3.8 / 4.6	0.80 / 1.03	0.94 / 1.2	2.7 / 2.6	2.2 / 2.3	2.1 / 1.8	1.9 / 2.4
PF weakness							
40% PF strength	2.4 / 2.9	0.29 / 0.46	1.05 / 1.2	0 / 0	0 / 0	0 / 0	0 / 0
60% PF strength	3.5 / –	0.78 / –	0.99 / –	1.7 / –	1.1 / –	1.8 / –	1.7 / –
90% PF strength	5.2 / 3.8	0.88 / 1.29	1.01 / 1.2	3.1 / 3.0	2.8 / 2.8	2.2 / 2.8	2.6 / 2.5
Passive stiffness							
20% of normal	4.6 / 4.4	1.14 / 1.63	0.95 / 1.2	3.0 / 2.8	2.8 / 2.7	2.3 / 2.4	2.7 / 2.4
50% of normal	4.4 / –	1.09 / –	0.94 / –	2.8 / –	2.5 / –	2.6 / –	2.4 / –
150% of normal	4.2 / –	0.59 / –	0.99 / –	2.6 / –	2.1 / –	2.8 / –	2.9 / –
200% of normal	3.5 / 4.8	0.67 / 0.30	0.94 / 1.2	2.4 / 2.0	1.3 / 2.4	2.7 / 2.0	2.4 / 1.9
Body mass							
+ 10% BM	4.4 / –	0.74 / –	0.94 / 1.2	3.0 / –	2.7 / –	2.5 / –	2.5 / –
+ 30% BM	4.3 / 7.1	0.70 / 1.51	0.88 / 1.2	3.3 / 3.4	3.0 / 3.4	1.9 / 0.4	2.7 / 2.7
Walking speed							
0.75 m/s	3.0	0.41	0.75	2.9	2.6	2.4	3.1
1.0 m/s	4.3	0.65	1.00	2.6	2.1	2.4	2.4
1.2 m/s	4.6	1.03	1.20	2.6	2.3	1.8	2.4

*AFO* ankle–foot orthosis, *BM* body mass, *PF* plantarflexor

## Discussion

Although simulations of walking without the AFO were overestimating the effects of plantarflexor weakness, our simulation framework predicted most effects of an AFO on lower limb kinematics and kinetics in bilateral plantarflexor weakness. Using this simulation framework, we showed a strong and interactive effect of severity of plantarflexor weakness and speed on the optimal AFO stiffness for walking energy cost reduction. Body mass and passive plantarflexor stiffness influenced the optimal stiffness to a less extent. Further, the optimal AFO stiffness for energy cost minimization was higher compared to the stiffnesses best normalizing specific gait parameters.

The simulations of walking without the AFO overestimated the effect of plantarflexor weakness regarding the maximal ankle dorsiflexion and external knee moment compared to experimental data, and were not as well predicted as with the AFO. Therefore, there is concern regarding the validity of walking energy cost and hence the effect sizes found by the different AFO stiffness levels. Despite that walking without AFO did not match experimental data well, the match for knee angle and moment was better compared to previous work simulating gait in bilateral plantarflexor weakness [22, 41]. Unlike these simulations, we did demonstrate persistent knee flexion and an external knee flexion moment commonly seen in these patients [2, 5], although to a larger extent as found in the experiments. The better knee flexion is likely explained by engaging the knee ligament at 10 degrees flexion instead of 5 degrees, which reduced the tendency of the model to walk with knee hyperextension as often found in predictive simulations [27, 42]. Furthermore, the simulations without AFO demonstrated more dorsiflexion during stance and no drop foot during swing, compared to the experimental data. The exaggerated dorsiflexion might be due to an underestimation of the passive ankle stiffness, as we kept this the same compared to the healthy model while patients may have a higher passive stiffness. The absence of a dropfoot in the simulations is due to the fact that we did not model dorsiflexor weakness to reduce the complexity of the simulations, while such weakness was present in our experimental population. Additionally, in previous work walking speed tended to be slow due to on the inclusion of all muscle activations squared in the cost function [22, 27]. Therefore, this factor was replaced by a penalty on activations above 50% of maximum for the Soleus and Gastrocnemius, as humans only tend to minimize energy cost when activations are kept relatively low [43]. This change in cost function increased simulated walking speed while unrealistic compensatory activations were avoided, and none of the muscle activations exceeded 50%. We modelled the AFO as a massless rotational spring around the ankle [44], which has been proven valid in inverse dynamic simulations [44, 45], but neglects the effects of AFO mass and of footplate stiffness on gait [46, 47]. We now demonstrated that such models are capable of predicting the effects of the AFO on lower leg kinematics and kinetics, although prediction of the ankle and hip power matched less well with experimental data [17]. The simulations predicted a slight reduction in ankle power with AFO instead of a slight increase. This may be explained by the lower predicted increase in walking speed with AFO in the simulations compared to the experimental data, and ankle power and AFO loading depend on walking speed [21, 48]. The higher hip power of the simulations has previously been reported with reflex-based controllers, and may be inherent to the used controller and not to modelling of the AFO [30, 42]. Despite the discrepancies between the simulations and experimental data, in general the simulations did capture most important gait deviations caused by plantarflexor and effects of the AFO that influence walking energy cost. Additionally, for most models the optimal AFO stiffness reduces the walking energy cost with 0.8–1.2 J/kg/m compared to the no AFO simulations, which is similar to the effect of AFOs with an optimized stiffness found experimentally [17]. This provides confidence that these simulations indeed provide an indication of the potential benefit of AFOs.

The simulations with different musculoskeletal models revealed that mainly severity of weakness, body mass and walking speed explained the experimentally found variety in individual optimal AFO stiffness in individuals with plantarflexor weakness [15, 17, 20]. In most cases, the optimal stiffness for energy cost minimization was between 3.0 and 5.0 Nm/degree with outliers as high as 7 Nm/degree for heavy models walking fast. This stiffness range coincides with the ranges reported in patients with neuromuscular diseases [15] and stroke [19]. That more severe weakness resulted in a higher optimal stiffness is in correspondence with data indicating that more affected patients put a larger strain on the AFO [21]. A larger strain on the AFO likely also explains why walking faster results in a higher optimal stiffness, although in previous hip torque-driven simulations this effect was much smaller [18]. The fact that these inverted-pendulum simulations neglected the effect of speed on knee flexion in the loading response [48], and the corresponding higher plantarflexor activation in early stance [49], may be a reason for the discrepancy in results. In case of plantarflexor weakness, the higher plantarflexor activation in the loading response is compensated for by the AFO, and hence more assistance is needed at higher speeds to minimize energy cost. This is in agreement with findings in healthy subjects where more assistance of an exoskeleton was needed at higher speeds to minimize energy cost [50]. Additionally, in our study, an interaction between the different factors studied seems to exist, as for example no effect of body mass on optimal stiffness was found when a higher, fixed walking speed was enforced. Without enforcing a higher walking speed, an increase in body mass resulted in a lower speed, which is more economic for obese people [51], which in turn reduced the optimal stiffness. Also, for passive stiffness an interaction with walking speed existed, as the effects of passive stiffness reduced when enforcing a faster speed. Additionally, it is noteworthy that contrary to the other models, for the model with 90% weakness the optimal stiffness increased at an enforced faster speed of 1.2 m/s. In this particular model, at low stiffness levels an external knee extension moment in the loading response was predicted, which may be favourable for energy cost, but will cause joint pain in humans [27, 52]. Additionally, the plantarflexors may be too weak to maintain a normal walking pattern at these faster speeds. Clinically, patients with severe bilateral plantarflexor weakness have a slower self-selected walking speed even with optimized AFOs [17, 20].

All models walking with optimal AFO stiffness walked with a relatively similar ankle moment and knee moment. The stiffness necessary to normalize joint angles required less stiff AFOs compared to normalization of the joint moments or energy cost, especially for the knee (Fig. 4). This corresponds with experimental findings in cerebral palsy [53] and polio survivors [16]. Normalization of the knee moment might be more directly related with quadriceps activation, and hence energy cost, than knee angle [54]. Additionally, to increase walking speed an increase in ankle moment is necessary, which apparently requires a higher stiffness compared to normalization of the ankle angle. However, the optimal AFO stiffness for energy cost minimization was consistently higher compared to the stiffness best normalizing both joint moments and angles, indicating that other factors also influence energy cost. Potentially, higher stiffness levels reduce energy cost further by taking over work of the plantarflexor muscles [10, 55] or by generating an external knee extension moment earlier in the gait cycle.

This is the first study using advanced forward musculoskeletal simulations to gain insights into the effect of pathophysiological muscle changes, body mass and walking speed on the optimal AFO stiffness. We extensively validated our model against patient data walking with and without AFO and found, despite uncertainties about the optimization criteria used by humans (cost function) [27] and limitations of our controller and 2D model, that most effects of AFOs were captured by the simulations. Nevertheless, the use of a 2D model may have changed the effect of the various factors on the optimal stiffness for energy cost minimization as mediolateral balance is ignored. In healthy gait, the plantarflexors contribute to mediolateral balance which accounts for approximately 10% of energy cost [56]. Use of an AFO also improves mediolateral balance, although differences in balance between stiffness levels are only marginal compared to the initial effect of an AFO [57]. Consequently, ignoring these benefits by using our 2D models may have influenced walking energy cost predictions and hence the selection of the optimal stiffness. Despite these shortcomings, the outcomes of our study may be insightful for clinicians that aim to match AFO stiffness towards the individual patient in case of bilateral plantarflexor weakness. Currently, off the shelf AFOs with a relatively low stiffness up to 3 Nm/degree are commonly provided [7, 58], while our simulations indicated that such stiffness levels are too low to normalize the joint kinematics and kinetics in most models and are not optimal for reducing walking energy cost. Therefore, we recommend to provide custom-made carbon AFOs as such AFOs allow for individualization of the AFO stiffness and—if needed- can be made with a stiffness above 3 Nm/degree. In the future, we aim to further develop our framework to predict patient-specific optimal stiffness’s by creating personalized models which account for the interaction between muscle pathophysiology, body mass and walking speed. Moreover, to predict the optimal stiffness for the individual as accurate as possible, we will consider other physiological factors such as proximal muscle strength, segment lengths and mass distribution. We expect these factors to have minor influence on the optimal stiffness for energy cost minimization, although combined may make a difference for the individual. Furthermore, by creating models varying in these factors an in-silico experiment can be conducted to create a large dataset of individual models. With such a dataset a regression analysis can be performed to create a selection algorithm for the optimal AFO, which would implementing AFOs with an optimal stiffness in clinical care easier. Besides efforts to implement AFO individualization in usual care, future research should focus on expanding our framework to study the effect of other AFO properties and expanding the model to study the effects of AFOs in other clinical populations such as stroke and cerebral palsy.

In conclusion, our forward simulations demonstrate that more severe plantarflexor weakness and faster walking speed can double the AFO stiffness aiming for minimal walking energy cost in individuals with bilateral plantarflexor. Passive muscle stiffness and body mass also noticeable influence the optimal stiffness, although to a lesser extent. These are an important step towards better matching the AFO stiffness to the individual user, although, before clinical implementation improved predictions of gait are required as currently the match with experimental data is insufficient. Future research should focus on predicting the optimal stiffness based on these characteristics using individualized models.

### Supplementary Information

**Additional file 1:** Model property settings.

**Additional file 2:** Difference between OpenSim and HyFyDy models.

**Additional file 3:** Simulation results without AFO.

**Additional file 4:** SPM results of simulation without AFO and AFO of 2.6 Nm/degree.

## Data Availability

The datasets used and/or analyzed during the current study are available from the corresponding author on reasonable request.
