# Supplementary material for: The interaction between muscle pathophysiology, body mass, walking speed and ankle foot orthosis stiffness on walking energy cost: a predictive simulation study
Source: J Neuroeng Rehabil. 2023 Sep 7;20:117. doi: 10.1186/s12984-023-01239-z (PMC10483766; doi:10.1186/s12984-023-01239-z)
Supplement: Supplementary file 1 — Additional file 1: Model property settings. [file 12984_2023_1239_MOESM1_ESM.pdf]

Appendix A

Table. Muscle parameters for healthy model without weakness

Muscle	Maximal isometric force (N)	Optimal fiber length (m)	Tendon slack length (m)	Pennation angle (radians)
**Semimembranosus**	2594	0.109	0.31	0
**Biceps Femoris Short Head**	1122	0.173	0.071	0.401
**Gluteus Maximus**	4759	0.147	0.127	0.000
**Iliopsoas**	5148	0.100	0.163	0.140
**Vastus Intermedius**	4530	0.087	0.136	0.052
**Rectus Femoris**	1169	0.114	0.305	0.087
**Gastrocnemius Medialis**	2241	0.060	0.390	0.297
**Soleus**	7147	0.050	0.250	0.436
**Tibialis Anterior**	1597	0.098	0.223	0.087

Table. Location and properties of the Hunt&Crossley contact spheres

	Heel-sphere (calcaneus as parent)	Toe-sphere (calcaneus as parent)
**x-position in parent in m**	0	0.185
**y-position in parent in m**	0.015	0
**z-position in parent in m**	0.005	0
**Radius in m**	0.03	0.03
**Stiffness**	2E6	2E6
**dissipation**	1	1
**Static/dynamic/viscous friction**	0.9	0.9
