# Supplementary material for: The interaction between muscle pathophysiology, body mass, walking speed and ankle foot orthosis stiffness on walking energy cost: a predictive simulation study
Source: J Neuroeng Rehabil. 2023 Sep 7;20:117. doi: 10.1186/s12984-023-01239-z (PMC10483766; doi:10.1186/s12984-023-01239-z)
Supplement: Supplementary file 2 — Additional file 2: Difference between OpenSim and HyFyDy models. [file 12984_2023_1239_MOESM2_ESM.pdf]

Appendix B

Hyfydy vs OpenSim

HyFyDy differs from OpenSim models in several ways, which may influence the simulation outcomes. In this appendix, an overview of all differences between the HyFyDy and OpenSim models is provided, and a comparison between simulation outcomes for healthy gait between the models is made.

Differences between HyFyDy and OpenSim

Joint constraint force

In OpenSim joint constraint forces are enforced automatically as generalized coordinates to describe the positions and velocities of the articulated bodies are used. In Hyfydy, each body has six degrees of freedom, and joint constraints are enforced explicitly.

Joint limit force

In OpenSim the damping of the joint limit force is constant after the set threshold, while in Hyfydy a non-linear damping, which is linearly proportional to the limit force, is used.

Muscle force

The implementation of the Millard Equilibrium Muscle Model [1] in Hyfydy differs from the OpenSim implementation in two ways:

1. The curves that describe the force-length and force-velocity relationships, as well as the curves for passive tendon and muscle forces, are defined through polynomials instead of splines as in OpenSim.

2. The muscle damping forces are computed explicitly instead through the iterative method in the original OpenSim implementation.

Integration

OpenSim and Hyfydy both implement several variable-step integrators with user-configurable error control. In Hyfydy, the accuracy criterium is based on the highest error for each body, while OpenSim uses a weighted sum of errors to determine accuracy.

Differences in optimization outcome

To test how these differences affected the simulation outcomes we simulated healthy gait using the same controller and cost function six times for both the OpenSim model and HyFyDy model. The most notable difference in simulation outcome was an external knee flexion moment in the loading response with the HyFyDy model, while the simulations with the OpenSim model walked with a neutral knee moment (see Appendix Figure).

1. Millard M, Uchida T, Seth A, Delp SL. Flexing computational muscle: modeling and simulation of musculotendon dynamics. Journal of biomechanical engineering. 2013;135(2):021005.

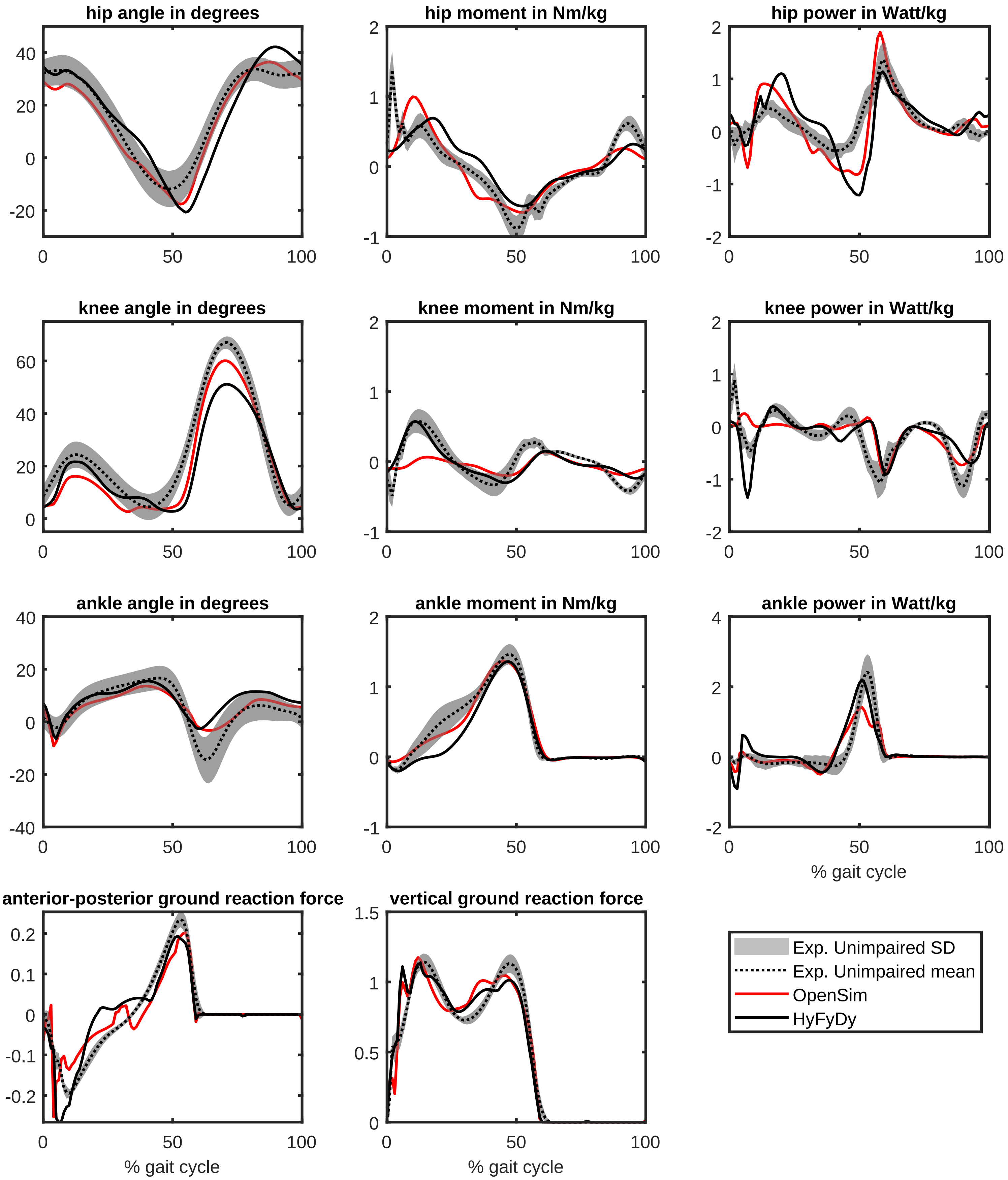

hip angle in degrees
hip moment in Nm/kg
hip power in Watt/kg
knee angle in degrees
knee moment in Nm/kg
knee power in Watt/kg
ankle angle in degrees
ankle moment in Nm/kg
ankle power in Watt/kg
anterior-posterior ground reaction force
vertical ground reaction force
% gait cycle
Exp. Unimpaired SD
Exp. Unimpaired mean
OpenSim
HyFyDy
