# Supplementary figures and images for: The interaction between muscle pathophysiology, body mass, walking speed and ankle foot orthosis stiffness on walking energy cost: a predictive simulation study
Source: J Neuroeng Rehabil. 2023 Sep 7;20:117. doi: 10.1186/s12984-023-01239-z (PMC10483766; doi:10.1186/s12984-023-01239-z)

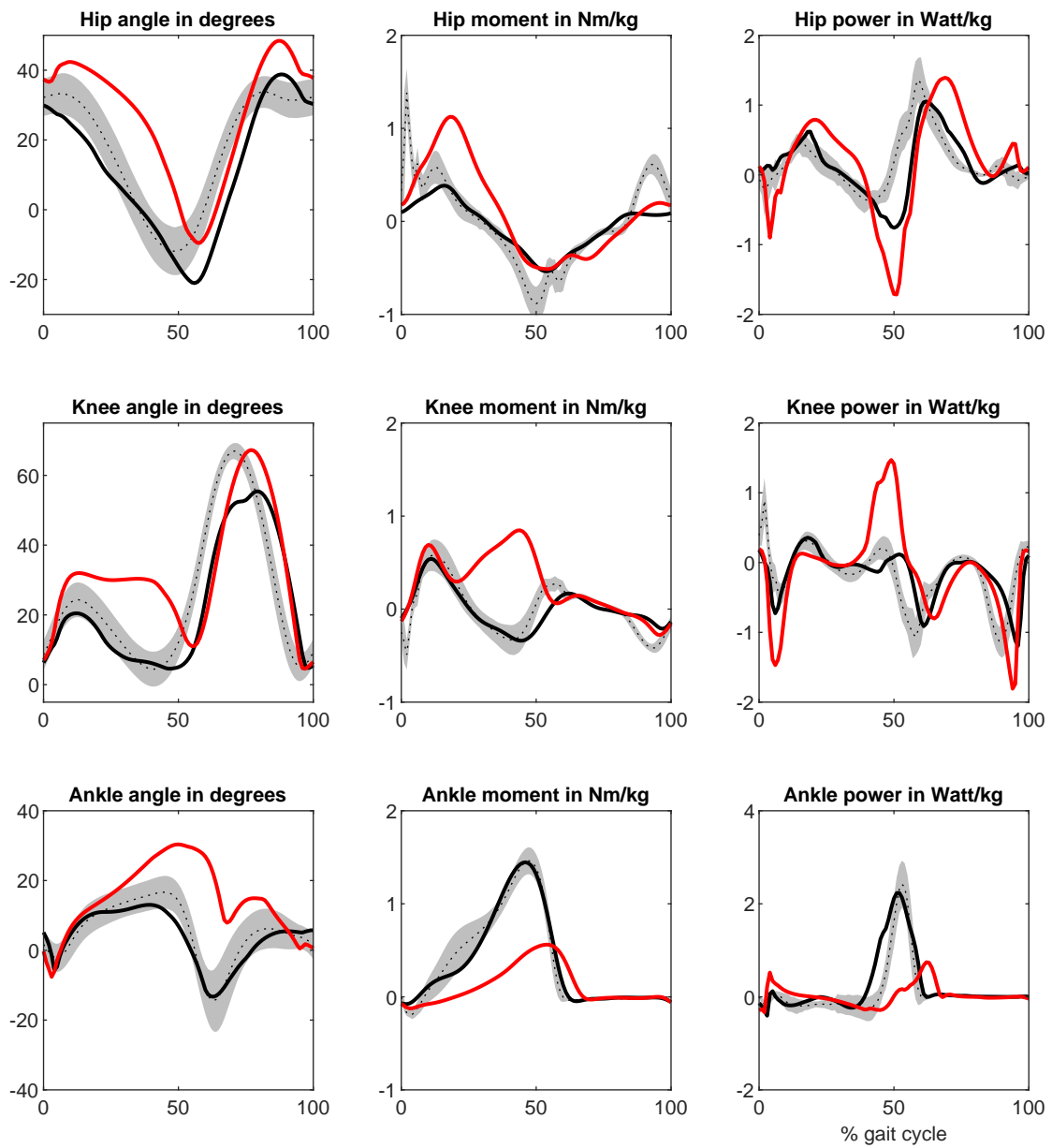

Supplement: Supplementary file 3 — Additional file 3: Simulation results without AFO. [file 12984_2023_1239_MOESM3_ESM.pdf]

## SPM results no AFO

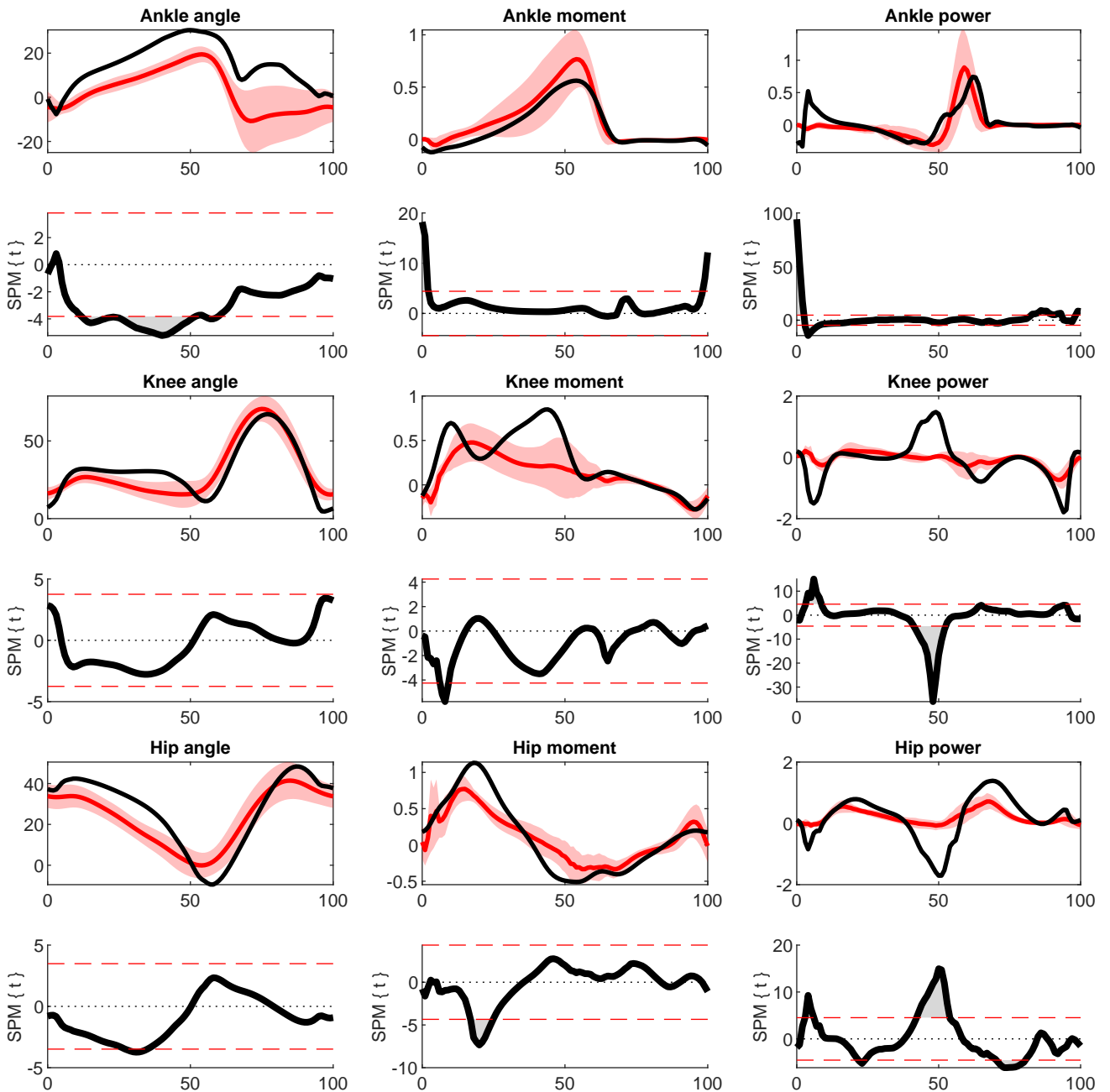

SPM results with AFO (2.6 Nm/degree)

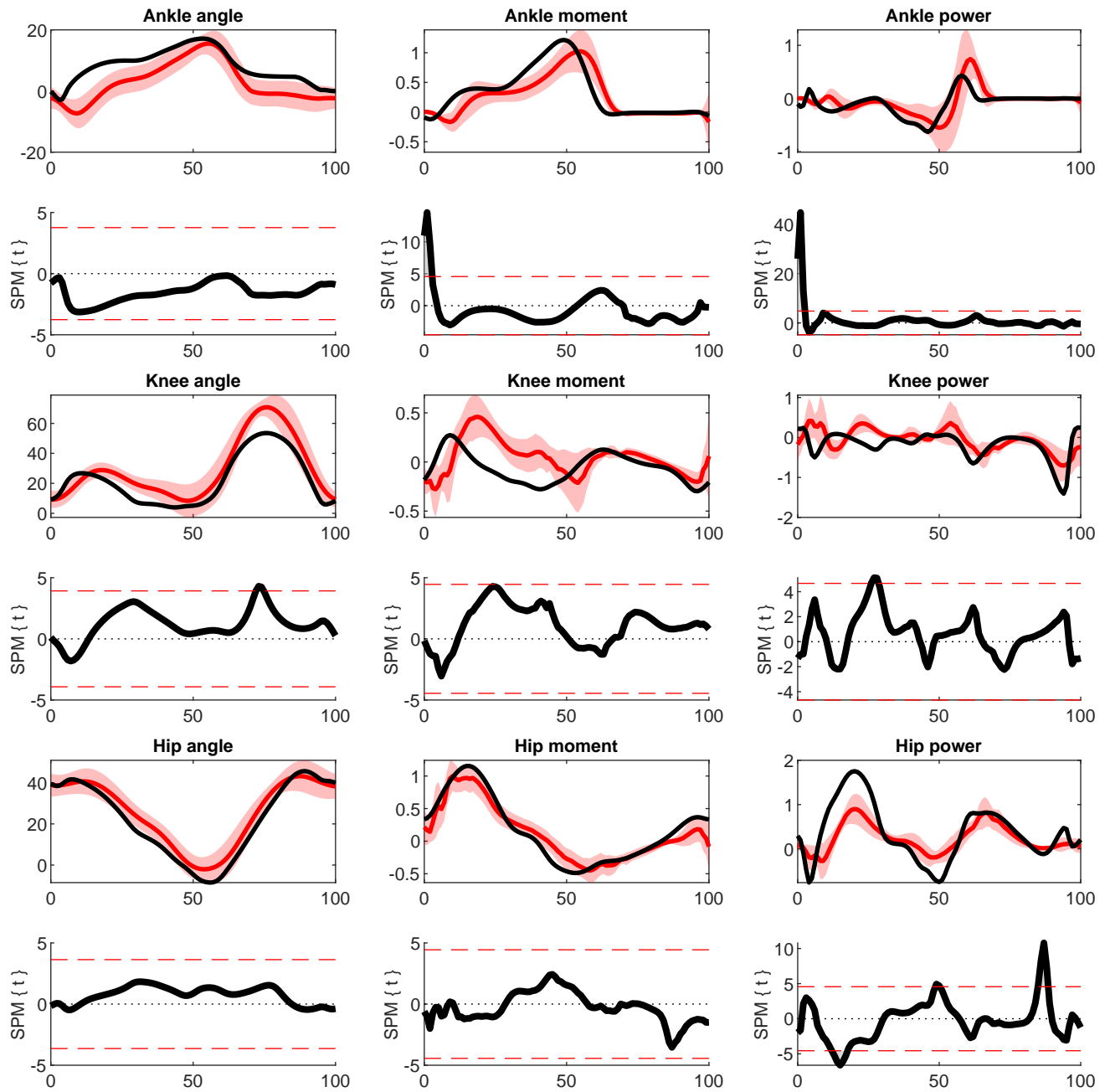

Supplement: Supplementary file 4 — Additional file 4: SPM results of simulation without AFO and AFO of 2.6 Nm/degree. [file 12984_2023_1239_MOESM4_ESM.pdf]
